# Beta-PSMC: uncovering more detailed population history using beta distribution

**DOI:** 10.1186/s12864-022-09021-6

**Published:** 2022-11-30

**Authors:** Junfeng Liu, Xianchao Ji, Hua Chen

**Affiliations:** 1 CAS Key Laboratory of Genomic and Precision Medicine, Beijing Institute of Genomics, Chinese Academy of Sciences, Beijing, 100101 China; 2 China National Center for Bioinformation, Beijing, 100101 China; 3 School of Future Technology, University of Chinese Academy of Sciences, Beijing, 100049 China; 4 CAS Center for Excellence in Animal Evolution and Genetics, Chinese Academy of Sciences, Kunming, 650223 China

**Keywords:** Demography inference, Beta distribution, Sequentially Markov coalescent

## Abstract

**Background:**

Inferring the demographic history of a population is essential in population genetic studies. Though the inference methods based on the sequentially Markov coalescent can present the population history in detail, these methods assume that the population size remains unchanged in each time interval during discretizing the hidden state in the hidden Markov model. Therefore, these methods fail to uncover the detailed population history in each time interval.

**Results:**

We present a new method called Beta-PSMC, which introduces the probability density function of a beta distribution with a broad variety of shapes into the Pairwise Sequentially Markovian Coalescent (PSMC) model to refine the population history in each discretized time interval in place of the assumption that the population size is unchanged. Using simulation, we demonstrate that Beta-PSMC can uncover more detailed population history, and improve the accuracy and resolution of the recent population history inference. We also apply Beta-PSMC to infer the population history of Adélie penguin and find that the fluctuation in population size is contrary to the temperature change 15–27 thousand years ago.

**Conclusions:**

Beta-PSMC extends PSMC by allowing more detailed fluctuation of population size in each discretized time interval with the probability density function of beta distribution and will serve as a useful tool for population genetics.

**Supplementary Information:**

The online version contains supplementary material available at 10.1186/s12864-022-09021-6.

## Background

Population history and demographic inference is a fundamental question in population genetic studies [1]. Over the past few years, many methods have been developed to infer population history with genome-scale data. Some approaches are based on allele frequency spectrum (aka, site frequency spectrum (SFS)) [2–6], which use diffusion process and coalescent process to construct SFS under various population history. The methods on the framework of diffusion process need a predefined simplified population model to infer population history, which are not suitable for the estimation of demography under very complex scenarios. Although model-flexible, the existing methods on the framework of coalescent process assume that the population size remains constant during the coalescent time [2, 6]. The other approaches are based on sequential Markov coalescent (SMC) [7–10], which spatially model recombination and coalescent events, to reveal more detailed population history using some form of hidden Markov model (HMM). Since the states of latent variables in the HMM-SMC methods are coalescence times which are continuous and infinite, HMM-SMC methods discretize them by dividing coalescence-times into a finite number of time intervals and assume that the function $\uplambda \left(t\right)$, which is scaled to population size, is a constant in each time interval. The assumption is a simplified approximation, and the model can describe the complex population history accurately when the time intervals are sufficiently small. Increasing the number of time intervals and discretization points dramatically increases the computational burden and running time, and makes the computation intractable [11].

Here, we present a new method, Beta-PSMC, that extends PSMC by replacing the function $\uplambda \left(t\right)$ within each time interval with the probability density function of beta distribution, which has two positive shape parameters denoted by $\alpha$ and $\beta$. Beta-PSMC can model a wide variety of changes of population size in each discretized time interval, as the beta distribution has flexible shapes, including *J*-shape, reverse *J*-shape, *U*-shape and reverse *U*-shape. Furthermore, a constant is a specific case of the function $\uplambda \left(t\right)$ in Beta-PSMC when setting $\alpha =\beta =1$. Therefore, Beta-PSMC can elucidate fine population history by further providing unprecedented details within each discretized time interval.

To validate the performance of our method, we conducted evaluation on Beta-PSMC using simulated data. We demonstrated that Beta-PSMC can uncover more detailed population size changes compared with PSMC, especially for the recent population history. We also applied Beta-PSMC to the genome of Adélie penguins to infer their population history during the Last Glacial. The results showed that there was negative correlation between the fluctuation of population size and temperature change 15–27 thousand years ago.

## Results

**Fig. 1:**
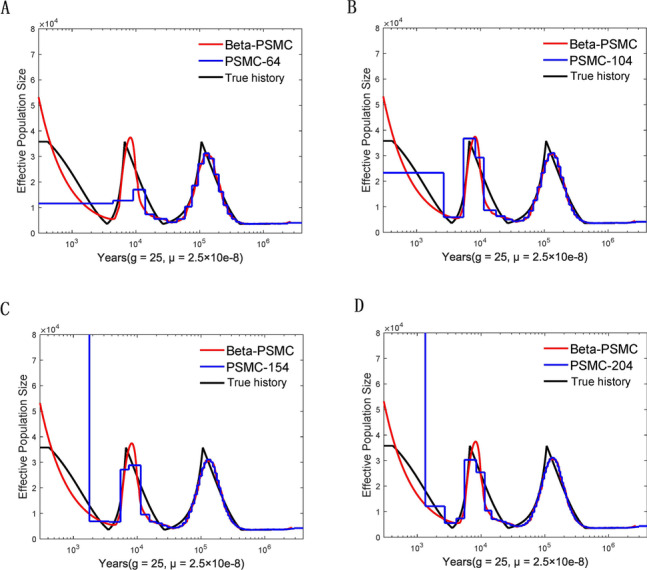
The inferred history of the simulated data from a population history with a series of population growth and decline. For Beta-PSMC, the number of discretized time intervals is 20 and the number of subintervals of each time interval is 3. **A** The number of discretized time intervals for PSMC is 64. **B** The number of discretized time intervals for PSMC is 104. **C** The number of discretized time intervals for PSMC is 154. **D** The number of discretized time intervals for PSMC is 204. *g,* generation time; *μ*, mutation rate

WE validated Beta-PSMC and compared it with PSMC with simulated data from a population history comprised of multiple epochs of population growths and declines (details in the Supplementary Materials). In order to scale results to real time, we assumed 25 years per generation and a mutation rate of 2.5 × 10^–8^ per generation per nucleotide [7]. The results in Fig. 1A showed that Beta-PSMC can recover the zigzag varying pattern of population size with a good resolution. Moreover, Beta-PSMC demonstrates better performance than PSMC in inferring recent population history (Fig. 1A-D). We also tried to improve the estimates of PSMC by refining discretization. Although PSMC has significantly better inference of population history from 3 thousand years ago (KYA) to the more distant past (Fig. 1B-D), the estimates within 2000 years remain poor (Fig. 1C-D).

Beta-PSMC subdivides each time interval into *k* subintervals for a given discretization, and employs two more parameters than PSMC. To compare the running time of Beta-PSMC and PSMC, we applied both methods to the same simulated data and repeated 10 times. When the number of time intervals is *n* and the number of subintervals is *k*, the average of running time of Beta-PSMC is close to that of PSMC with *n* × *k* intervals (Supplementary Table S1). Although Beta-PSMC with the same *n* is slower than PSMC, Beta-PSMC needs fewer time intervals when inferring population history with good resolution (Fig. 1).

We applied Beta-PSMC and PSMC to infer the population dynamics of Adélie penguin with the published genome sequence [12] (Supplementary Fig. S1). The population history of Adélie penguin between 100 and 10 KYA is of specific interest, since the population dynamics of Adélie penguin is hypothesized to be strongly influenced by the Antarctic climatic variation during the last glaciation [12]. In contrast with PSMC, Beta-PSMC uncovered more detailed population history in the period of 15–27 KYA; the effective population size of Adélie decreases gradually from about 20 KYA after increasing gradually from 27 KYA. This fluctuation in the period of 15–27 KYA is contrary to the trend of temperature change (Supplementary Fig. S1), indicating that the effective population size of Adélie penguin may be strongly affected by Antarctic climate. Hu et al. [13] reconstructed the population history of Adélie at Ross Island over the past 700 years by determining organic markers in a sediment profile and found that the population sizes of Adélie penguin were the highest in the Little Ice Age. Their conclusion that the population size of Adélie was the highest during a cold period is consistent with our inferred history during the last glaciation.

## Discussion

Beta-PSMC extends the PSMC method by splitting time intervals into subintervals to achieve higher accuracy in demographic inference, however, running time also increases with the number of subintervals (Supplementary Table S1). We analyzed the effect of subinterval numbers using simulated data by running Beta-PSMC with different subinterval numbers: *k* = 2, *k* = 3, *k* = 5, and *k* = 7 respectively. The results showed that the estimates were rough when *k* = 2 (Supplementary Fig. S2A) and the similar performance with good resolution could be achieved when *k* = 3, *k* = 5, and *k* = 7 (Supplementary Fig. S2B-D). This implies that a smaller subinterval number, e.g., *k* = 3 is sufficient for the accuracy of most demographic scenarios.

Another advantage of Beta-PSMC over PSMC is on the inference of recent demographic history. PSMC provides poor estimates of population sizes for recent history (< 10KYA) due to limited information of recombination and coalescence events during that time range from a single individual genome. The accuracy of estimates even declined with the increase of number of discretized time intervals when inferring the population history with one sharp bottleneck followed by an exponential growth within 10KYA (Supplementary Fig. S3A-D). This is due to the fact that refining discretization results in the decline of recombination events in each recent discretized time interval, which further reduces the power of PSMC. In order to increase the power of Beta-PSMC for inferring recent demographic history, we combine the first three discretized time intervals to accumulate more recombination events, and use the shape of the probability density function of beta distribution to allow for population size fluctuation during the time interval. The simulation results indicate that the strategy is valid (Supplementary Fig. S3E-F). Compared with PSMC, Beta-PSMC improves the inference accuracy and resolution for the recent population history by using the probability density function of beta distribution. However, the above strategy is not available for the recent population history with one sharp bottleneck followed by an instant growth (Supplementary Fig. S4A-B). Although more recombination events are helpful to increase the power of Beta-PSMC for inferring recent demographic history, there exists the instant change of population size in the combined discretized time interval. Regrettably, the shape of the probability density function of beta distribution is continuous and not available for the instant change of population size in the combined discretized time interval (Supplementary Fig. S4A). If the instant growth happened more early, the inference accuracy was improved due to the continuous population size in the combined discretized time interval (Supplementary Fig. S4B).

It should be emphasized that the probability density function of beta distribution in Beta-PSMC is used to approximate the varying population size in each discretized time interval based on the observed sequences, and is different from another smoothing step for the estimated population sizes from different time intervals. For each discretized time interval, there are five main types of the fluctuation of population size: gradual growth, gradual decline, gradual growth after gradual decline, gradual decline after growth, and no fluctuation. The above five types can be described by the probability density function of beta distribution with two parameters, which has flexible shapes, such as *J*-shape, reverse *J*-shape, *U*-shape, reverse *U*-shape and straight line shape. Although a second-order polynomial can also be used to approximate the five types of the fluctuation of population size, there are three parameters in the second-order polynomial. The other type of spline, such as cubic spline and B-spline, can be used to approximate the more complicated fluctuation of population size, but there are more parameters to be estimated.

Although Beta-PSMC improves the performance of PSMC, it has three disadvantages. The first disadvantage is that Beta-PSMC failed to improve the inference accuracy for the instant change, especially in the recent population history (Supplementary Fig. S4A-B). This is because the shape of the probability density function of beta distribution is continuous and not available for the instant change of population size in the given time interval. The second disadvantage is that the curve of population size generated from Beta-PSMC is not smooth at the joint between adjacent time intervals (Supplementary Fig. S5A-B). This is due to the singular boundary point of each discretized time interval. In order to smooth the curve of population size, a quadratic curve fitting was used at the joint between adjacent time intervals (Supplementary Fig. S5C-D). The last disadvantage is that the choice of some parameters can significantly influence the estimation results. Firstly, the number of subintervals can affect the estimation results (Supplementary Fig. S2A-D). According to the simulation results, we advise to choose the number of subintervals of 3. Secondly, the pattern of parameter vectors can also affect the estimation results. In order to improve the estimation, we adopt the strategy as follows: Each discretized time interval is spanned by one parameter vector; If the estimated result is singular in one discretized time interval (Supplementary Fig. S3E), the discretized time interval is combined with adjacent discretized time intervals and then spanned by one parameter vector until the estimated result is no longer singular (Supplementary Fig. S3F).

## Conclusions

PSMC can infer the demographic history accurately using a single personal genome for a wide time range, serving as a very popular tool in population genetic studies. The Beta-PSMC method presented in this paper extends PSMC by allowing more detailed fluctuation of population size in each discretized time interval with the probability density function of beta distribution. This is especially useful for some scenarios that the population size fluctuates and thus improves the fine-scale inference of complex demographic history during some short time intervals; Furthermore, Beta-PSMC in some degree improves the accuracy and resolution for the recent population history inference. We expect Beta-PSMC to supplement PSMC towards a flexible tool for inferring population history using genomic data.

## Methods

### Beta-PSMC model

Beta-PSMC method is an extension of the widely used PSMC method [7]. It is different from PSMC in modeling the scaled population size in each discretized time interval when discretizing coalescence-times. PSMC sets $0\le {t}_{0}<{t}_{1}<\cdots <{t}_{n}<{t}_{n+1}=\infty$ and assumes the function $\uplambda \left(t\right)$, which is scaled to population size, is a constant ${\uplambda }_{i}\left(i=0,\cdots ,n\right)$ in each discretized time interval $\left[{t}_{i},\left.{t}_{i+1}\right)\right.$. Given a maximum of the most recent common ancestor (TMRCA) *T*_*max*_, PSMC sets the boundaries of discretized time intervals to be ${t}_{i}=0.1exp\left[{}^{i}\!\left/ \!{}_{n}\right.log\left(1+{T}_{max}\right)\right]-0.1, i=0,\cdots ,n$. For each discretized time interval $\left[{t}_{i},\left.{t}_{i+1}\right)\right.$ when $0\le i<n$, Beta-PSMC adopts a form of the function $\uplambda \left(t\right)$ as follows,

$$\uplambda \left(t\right)=f\left(\frac{t-{t}_{i}}{{t}_{i+1}-{t}_{i}};{\alpha }_{i},{\beta }_{i}\right)\times {\uplambda }_{i} (1)$$

where $f\left(x;{\alpha }_{i},{\beta }_{i}\right)$ is the probability density function of beta distribution and $\left({\alpha }_{i},{\beta }_{i}\right)$ are two shape parameters of beta distribution; $x=\frac{t-{t}_{i}}{{t}_{i+1}-{t}_{i}}$ and ${t}_{i}\le t<{t}_{i+1}$; ${\uplambda }_{i}$ is a constant. In the time interval $\left[{t}_{n},\left.{t}_{n+1}\right)\right.$, Beta-PSMC assumes the function $\uplambda \left(t\right)$ to be a constant ${\uplambda }_{n}$. In order to estimate the shape parameters of the beta distribution in each discretized time interval $\left[{t}_{i},\left.{t}_{i+1}\right)\right.$, $\uplambda \left(t\right)$ is discretized into ${\uplambda }_{i,j}\left(j=0,\cdots ,k-1\right)$ subintervals according to the following equation,

$${\lambda }_{i,j}=\frac{1}{{t}_{i+1,j}-{t}_{i,j}}{\int }_{{t}_{i,j}}^{{t}_{i+1,j}}\uplambda \left(t\right)dt (2)$$

where $${t}_{i,j}={t}_{i}+\frac{j}{k}\left({t}_{i+1}-{t}_{i}\right)$$ and $${t}_{i+1,j}={t}_{i}+\frac{j+1}{k}\left({t}_{i+1}-{t}_{i}\right), j=0,\cdots ,k-1$$.

Then, the *n* × *k* scaled population sizes, which are functions of the parameter vector $\left({{\uplambda }_{i},\alpha }_{i},{\beta }_{i}\right) 0\le i<n$, and ${\uplambda }_{n}$ are estimated by fitting the likelihood function to the observation data using the expectation–maximization (EM) algorithm similar to PSMC. Finally, the probability density function of beta distribution with the estimated parameters $\left({{\uplambda }_{i},\alpha }_{i},{\beta }_{i}\right) 0\le i<n$ is used to present the fluctuation of population size in the discretized time interval $\left[{t}_{i},\left.{t}_{i+1}\right)\right.$.

### Coalescent simulation

One hundred haploid sequences of 10 Mb were simulated in three scenarios. In each scenario, the number of samples is 100 and all sample size is one genome. In the first scenario, the population experiences a series of population growths and declines (Fig. 1). In the second scenario, a sharp bottleneck is followed by an exponential expansion (Fig. S3). In the third scenario, a sharp bottleneck is followed by an instant growth (Fig. S4). We assumed the generation time of 25 years. The neutral mutation rate was chosen to be 2.5 × 10–8 per generation per site. The program msHOT was used to generate the simulated data.

### User-specified parameter settings for Beta-PSMC

To improve the accuracy and resolution of demographic inference, blocks of adjacent discretized time intervals can be combined to have the same parameter vector via a user-specified pattern. When analyzing the simulated data from the first scenario, the setting for Beta-PSMC is ‘20*1’, which means each of the 20 parameter vectors spans one discretized time interval. For the simulated data from the second scenario, the setting is ‘1*3 + 17*1’, with the first parameter vector spanning the first three discretized time intervals and each of the next 17 parameter vectors for one discretized time interval. In addition, one discretized time interval or combined discretized time interval can also be divided equally into independent intervals, each of which is spanned by one parameter vector.

### Scaling to real time and population size

${\theta }_{0}=4{N}_{0}\mu$ Of Beta-PSMC, which is similar to that of PSMC, is the scaled mutation rate, where $\mu$ is the point mutation rate. The estimated TMRCA is in units of $2{N}_{0}$ generations, and $\uplambda \left(t\right)$ is scaled to ${N}_{0}$ as well. The mutation rate should be specified to estimate ${N}_{0}={\theta }_{0}/4\mu$. To convert generations to years, the generation time is specified.

### Smoothing fits

In each discretized time interval, the curve of population size generated from Beta-PSMC is described by the probability density function of beta distribution with two inferred shape parameters. However, the connections of curves between adjacent time intervals are not smooth (Supplementary Fig. S5A-B). In order to smooth curves among these time intervals, 5% from the left side of the curve of population size and 10% from the right side are discarded and then a quadratic curve fitting was used to connect the curves of population size between adjacent time intervals (Supplementary Fig. S5C-D). The curve of population size was defined in the interval [0,1]. The 5% from the left side of the curve means the interval [0,0.05] and the 10% from the right means the interval (0.9,1). Three selected points of two adjacent intervals, two of which are at 0.8 and 0.9 of the previous interval and the last is at 0.05 of the next interval, are used to quadratic curve fitting.

### Read alignment and calling the consensus sequence

Adélie penguin genomic data was obtained from the NCBI Sequence Read Archive (SRR1145007). These sequence reads were mapped by Bowtie2 [14] against the Adélie penguin reference genome [15]. The diploid consensus sequence was obtained using the ‘pileup’ command of the SAMtools software package [16]. The commands are in the Supplementary Materials.

## Supplementary Information

**Additional file 1:** **Table S1.** The running times ofBeta-PSMC and PSMC on the simulation data based on the population history witha series of population growths and declines. Note. For Beta-PSMC, thenumber of discretized time intervals is 10. “*n*=30; k=3”: the number ofdiscretized time intervals is 30 for PSMC and the number of subintervals foreach time interval is 3. The unit of running time is minute. The number ofrepeats is 10. **Fig.S1.** Population sizes through time inferred from Adélie penguin genomesequences. The data of temperature change is from Li et al. (2014). g,generation time; μ, mutation rate. **Fig. S2.** The population history inferred with Beta-PSMCwith different subinterval settings for a simulated data from a population witha series of growths and declines. (A) The number of subintervals for each timeinterval is 2. (B) The number of subintervals for each time interval is 3. (C)The number of subintervals for each time interval is 5. (D) The number ofsubintervals for each time interval is 7. g, generation time; μ, mutation rate. **Fig. S3.** The population history inferred with PSMC and Beta-PSMC withdifferent settings for a simulated data from a population with one sharpbottleneck followed by an exponential expansion. For Beta-PSMC, the number of subintervalsfor each time interval is 3. (A) The number of discretized time intervals is 20for PSMC and the user-specified pattern is “20*1”. (B) The number ofdiscretized time intervals is 30 for PSMC and the user-specified pattern is“30*1”. (C) The number of discretized time intervals is 40 for PSMC and theuser-specified pattern is “40*1”. (D) The number of discretized time intervalsis 50 for PSMC and the user-specified pattern is “50*1”. (E) The number ofdiscretized time intervals is 20 for Beta-PSMC and the user-specified patternis “20*1”. (F) The number of discretized time intervals is 20 for Beta-PSMC andthe user-specified pattern is “1*3+17*1”. g, generation time; μ, mutation rate. **Fig. S4.** The population history inferred with PSMC and Beta-PSMC for asimulated data from a population with one sharp bottleneck followed by an instantgrowth. For Beta-PSMC, the number of subintervals for each time interval is 3. Thenumber of discretized time intervals is 20 for Beta-PSMC and the user-specifiedpattern is “1*4+16*1”. The number of discretized time intervals is 64 for PSMCand the user-specified pattern is “4+25*2+4+6”. g, generation time; μ, mutation rate. (A) The instant growth happened at 20thousand years ago (KYA). (B) The instant growth happened at 80KYA. **Fig. S5.** Smoothing the connections between adjacent timeintervals to improve the inference of demographic history. g, generation time; μ, mutation rate. The number of subintervals for each timeinterval is 3.

## Data Availability

Beta-PSMC is implemented in C and is available under the MIT License. The source code and documentation are available at https://github.com/chenh-big/Beta-PSMC. The genomic data of Adélie penguin are from the NCBI (https://www.ncbi.nlm.nih.gov/sra/) under accession number SRR1145007.

## Abbreviations

SMC: Sequentially Markovian Coalescent
PSMC: Pairwise Sequentially Markovian Coalescent
HMM: Hidden Markov Model
