# Supplementary material for: Beta-PSMC: uncovering more detailed population history using beta distribution
Source: BMC Genomics. 2022 Nov 30;23:785. doi: 10.1186/s12864-022-09021-6 (PMC9710181; doi:10.1186/s12864-022-09021-6)
Supplement: Supplementary file 1 — Additional file 1: Table S1. The running times ofBeta-PSMC and PSMC on the simulation data based on the population history witha series of population growths and declines. Note. For Beta-PSMC, thenumber of discretized time intervals is 10. “n=30; k=3”: the number ofdiscretized time intervals is 30 for PSMC and the number of subintervals foreach time interval is 3. The unit of running time is minute. The number ofrepeats is 10. Fig.S1. Population sizes through time inferred from Adélie penguin genomesequences. The data of temperature change is from Li et al. (2014). g,generation time; μ, mutation rate. Fig. S2. The population history inferred with Beta-PSMCwith different subinterval settings for a simulated data from a population witha series of growths and declines. (A) The number of subintervals for each timeinterval is 2. (B) The number of subintervals for each time interval is 3. (C)The number of subintervals for each time interval is 5. (D) The number ofsubintervals for each time interval is 7. g, generation time; μ, mutation rate. Fig. S3. The population history inferred with PSMC and Beta-PSMC withdifferent settings for a simulated data from a population with one sharpbottleneck followed by an exponential expansion. For Beta-PSMC, the number of subintervalsfor each time interval is 3. (A) The number of discretized time intervals is 20for PSMC and the user-specified pattern is “20*1”. (B) The number ofdiscretized time intervals is 30 for PSMC and the user-specified pattern is“30*1”. (C) The number of discretized time intervals is 40 for PSMC and theuser-specified pattern is “40*1”. (D) The number of discretized time intervalsis 50 for PSMC and the user-specified pattern is “50*1”. (E) The number ofdiscretized time intervals is 20 for Beta-PSMC and the user-specified patternis “20*1”. (F) The number of discretized time intervals is 20 for Beta-PSMC andthe user-specified pattern is “1*3+17*1”. g, generation time; μ, mutation rate. Fig. S4. The population history [file 12864_2022_9021_MOESM1_ESM.docx]

Supplementary Material

**Beta-PSMC: Uncovering more detailed population history using beta distribution**

**1. Simulation Commands**

**-- the population history with a series of population growths and declines**

./msHOT-lite 2 100 -l -t 7156.0000000 -r 2000.0000 10000000 -eN 0 5 -eG 0.000582262 527.27 -eG 0.004949226 -527.27 -eG 0.00931619 82.3865 -eG 0.0372648 -20.5966 -eG 0.149059 5.14916 -eN 0.596236 0.5 > ms_out

./ms2psmcfa.pl ms_out > input_.psmcfa

**-- the population history with one sharp bottleneck followed by an exponential expansion**

./msHOT-lite 2 100 -l -t 27320 -r 4520 10000000 -eG 0 299.5732 -eN 0.01 0.05 -eN 0.0375 0.5 > ms_out

./ms2psmcfa.pl ms_out > input_.psmcfa

**-- the population history with one sharp bottleneck followed by an instant growth** ./msHOT-lite 2 100 -l -t 27320 -r 4520 10000000 -eN 0.01 0.05 -eN 0.0375 0.5 -eN 1.25 1 > ms_out

./ms2psmcfa.pl ms_out > input_.psmcfa

**2. Analyzing Commands**

**Beta-PSMC:**

./beta-psmc -N20 -t15 -r5 -p “20*1” –K1 –L3 -o output.psmc input.psmcfa (for simulation data based on the population history with a series of population growths and declines)

./beta-psmc -N20 -t15 -r5 -p “1*3+17*1” -o output.psmc input.psmcfa (for simulation data based on the population history with one sharp bottleneck followed by an exponential expansion )

./beta-psmc -N20 -t15 -r5 -p “1*4+16*1” -o output.psmc input.psmcfa (for simulation data based on the population history with one sharp bottleneck followed by an instant growth )

./beta-psmc –N30 -t15 -r5 -p “20*1” -o output.psmc input.psmcfa (for Adélie penguin data)

**PSMC:**

./psmc -N20 -t15 -r5 -p “64*1” (-p “104*1”, -p “154*1”, -p “204*1”) -o output.psmc input.psmcfa (for simulation data based on the population history with a series of population growths and declines)

./psmc -N20 -t15 -r5 -p “20*1” (-p “30*1”, -p “40*1”, -p “50*1”) -o output.psmc input.psmcfa (for simulation data based on the population history with one sharp bottleneck followed by an exponential expansion)

./psmc –N20 -t15 -r5 -p "4+25*2+4+6" -o output.psmc input.psmcfa (for simulation data based on the population history with one sharp bottleneck followed by an instant growth)

./psmc –N30 -t15 -r5 -p "4+25*2+4+6" -o output.psmc input.psmcfa (for Adélie penguin data)

**Bowtie2 and SAMtools:**

bowtie2 -build adelie.fa adelie

bowtie2 -p 5 -x adelie -1 input_1.fq -2 input_2.fq | samtools sort -O bam -o adelie.bam --threads 3

bcftools mpileup -C50 -f adeliae.fa adelie.bam | bcftools call -c -V indels | vcfutils.pl vcf2fq -d 10 -D 100 | gzip > adeliae.fq.gz

**3. Simulation Results**

**Table S1.** The running times of Beta-PSMC and PSMC on the simulation data based on the population history with a series of population growths and declines.

|  | Beta-PSMC (units: min) | PSMC (units: min) |
| --- | --- | --- |
| n=30；k=3 | 10.80±0.42 | 11.50±0.53 |
| n=50；k=5 | 25.90±0.74 | 29.20±0.79 |
| n=70；k=7 | 45.20±1.23 | 54.90±1.10 |

Note. For Beta-PSMC, the number of discretized time intervals is 10. “n=30; k=3”: the number of discretized time intervals is 30 for PSMC and the number of subintervals for each time interval is 3. The unit of running time is minute. The number of repeats is 10.


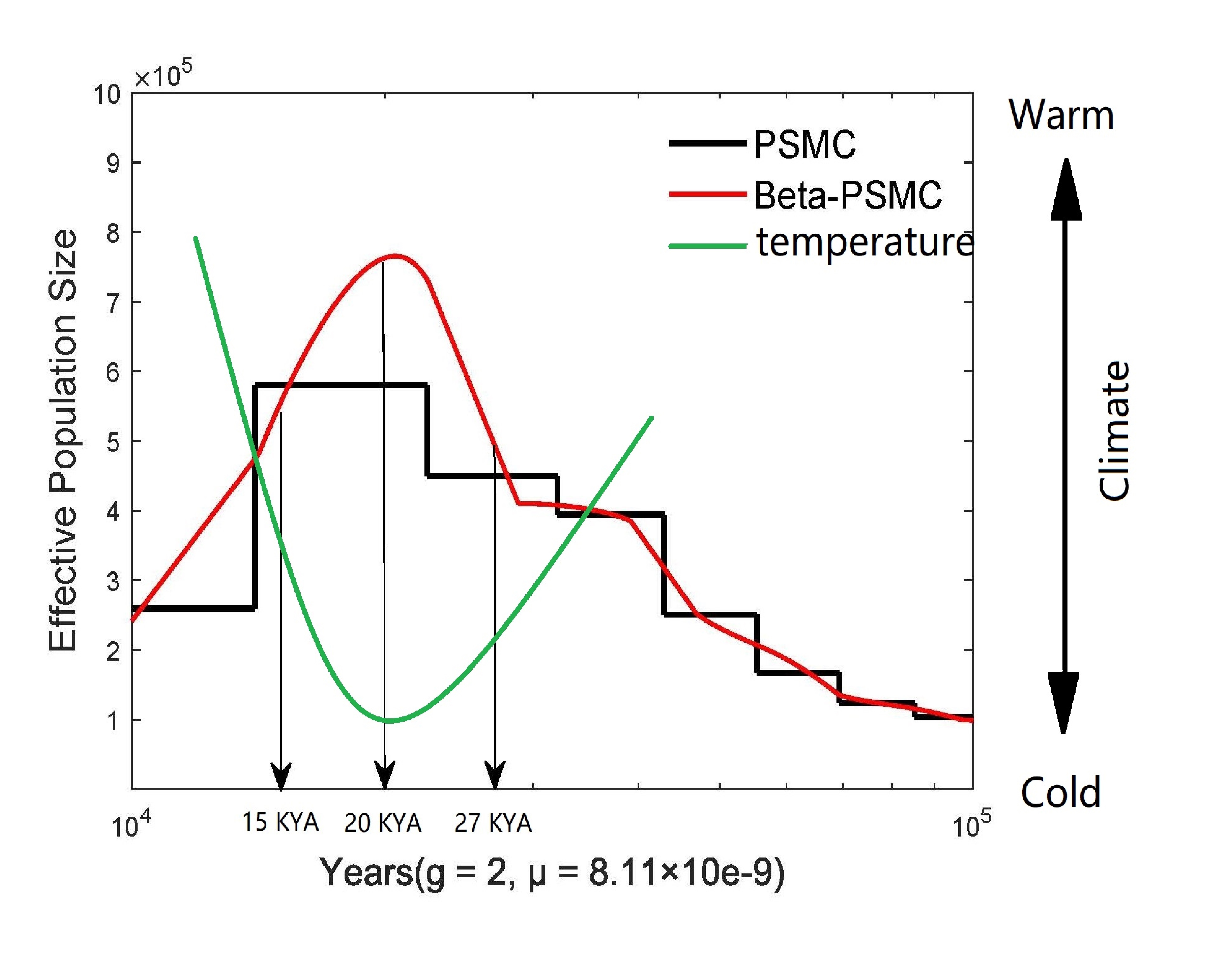


Fig. S1. Population sizes through time inferred from Adélie penguin genome sequences. The data of temperature change is from Li et al. (2014). g, generation time; μ, mutation rate.


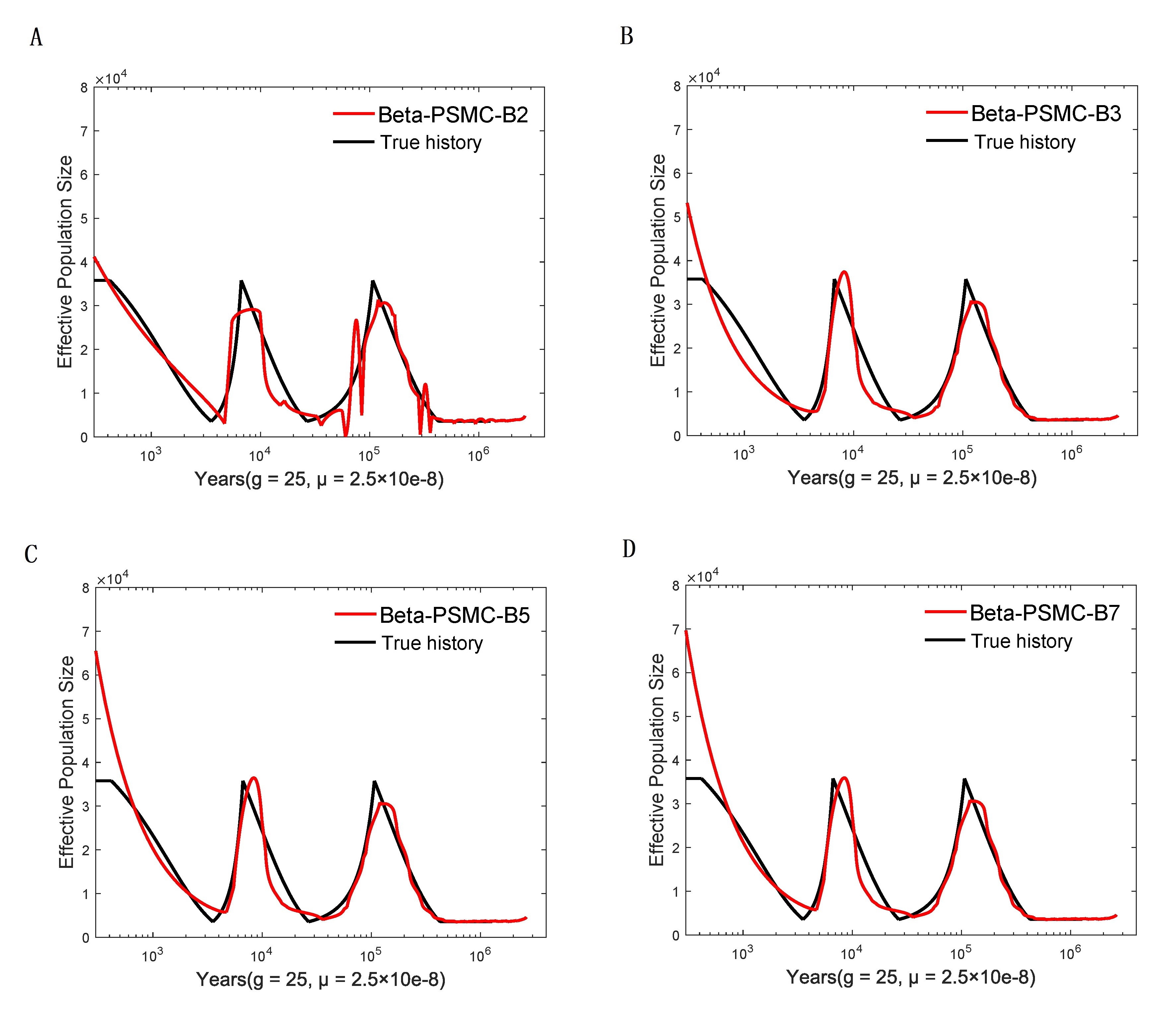


Fig. S2. The population history inferred with Beta-PSMC with different subinterval settings for a simulated data from a population with a series of growths and declines. (A) The number of subintervals for each time interval is 2. (B) The number of subintervals for each time interval is 3. (C) The number of subintervals for each time interval is 5. (D) The number of subintervals for each time interval is 7. g, generation time; μ, mutation rate.


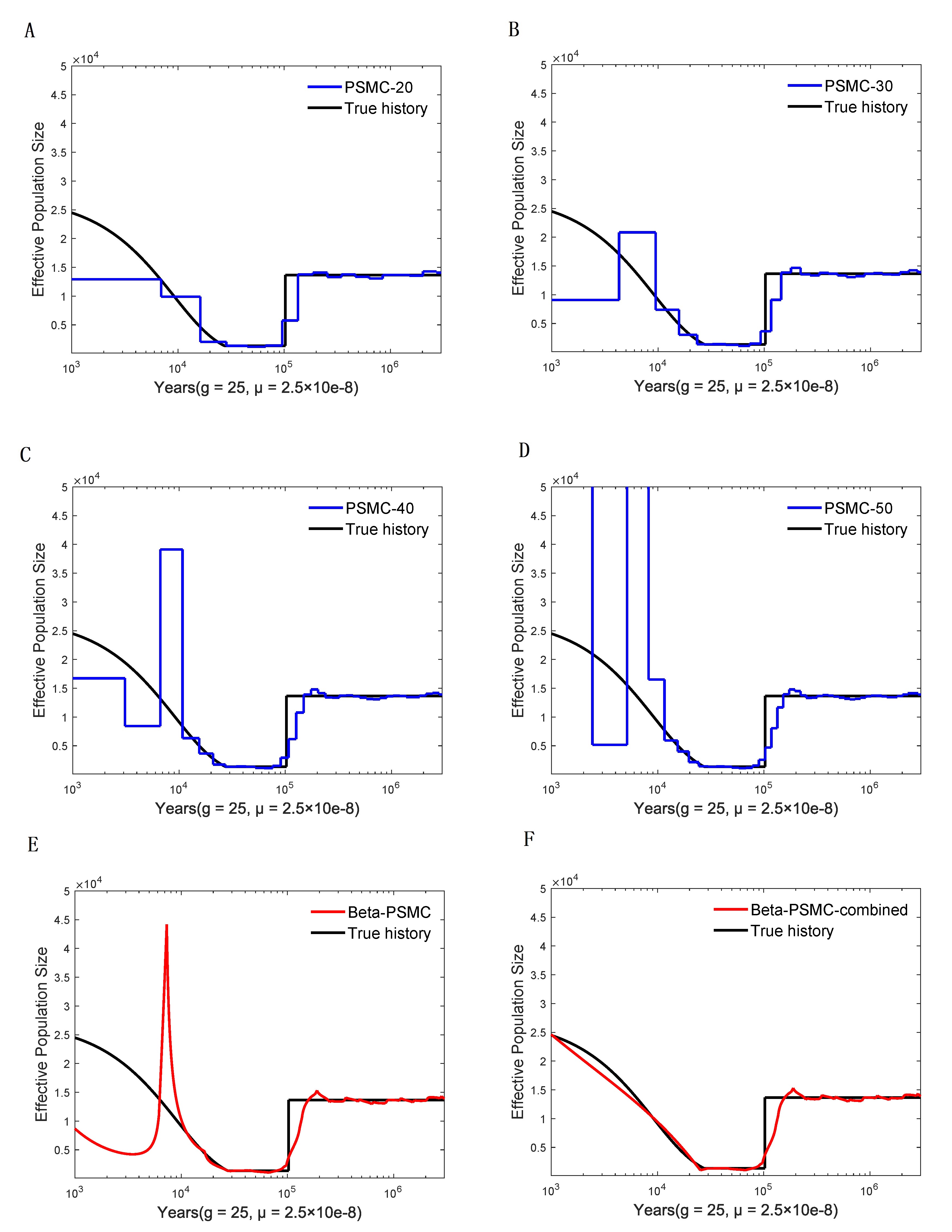


Fig. S3. The population history inferred with PSMC and Beta-PSMC with different settings for a simulated data from a population with one sharp bottleneck followed by an exponential expansion. For Beta-PSMC, the number of subintervals for each time interval is 3. (A) The number of discretized time intervals is 20 for PSMC and the user-specified pattern is “20*1”. (B) The number of discretized time intervals is 30 for PSMC and the user-specified pattern is “30*1”. (C) The number of discretized time intervals is 40 for PSMC and the user-specified pattern is “40*1”. (D) The number of discretized time intervals is 50 for PSMC and the user-specified pattern is “50*1”. (E) The number of discretized time intervals is 20 for Beta-PSMC and the user-specified pattern is “20*1”. (F) The number of discretized time intervals is 20 for Beta-PSMC and the user-specified pattern is “1*3+17*1”. g, generation time; μ, mutation rate.


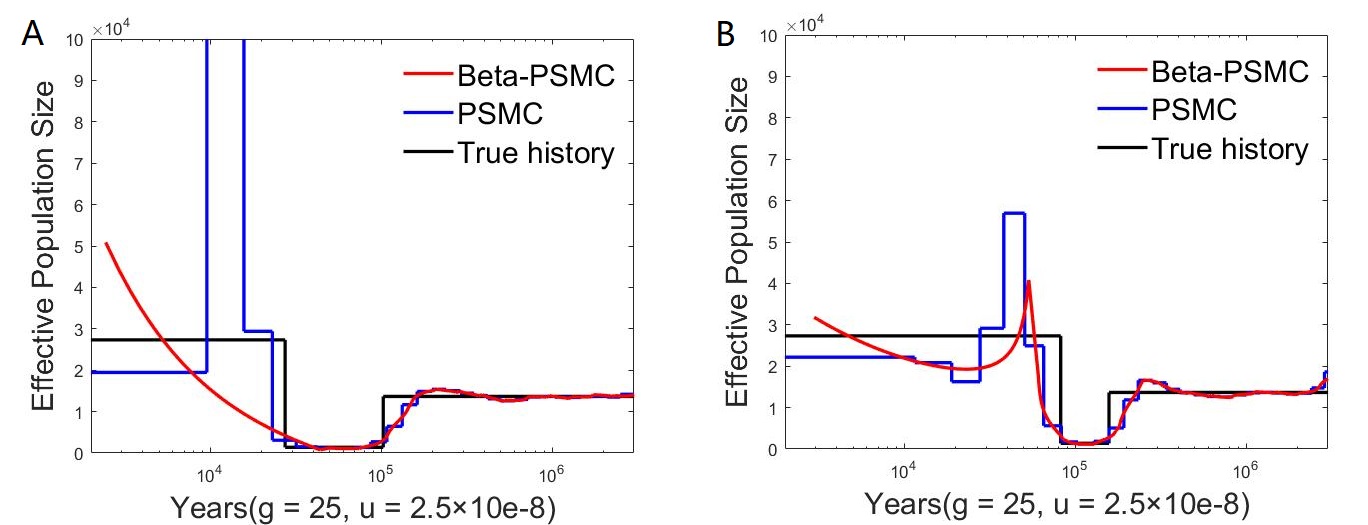


Fig. S4. The population history inferred with PSMC and Beta-PSMC for a simulated data from a population with one sharp bottleneck followed by an instant growth. For Beta-PSMC, the number of subintervals for each time interval is 3. The number of discretized time intervals is 20 for Beta-PSMC and the user-specified pattern is “1*4+16*1”. The number of discretized time intervals is 64 for PSMC and the user-specified pattern is “4+25*2+4+6”. g, generation time; μ, mutation rate. (A) The instant growth happened at 20 thousand years ago (KYA). (B) The instant growth happened at 80KYA.


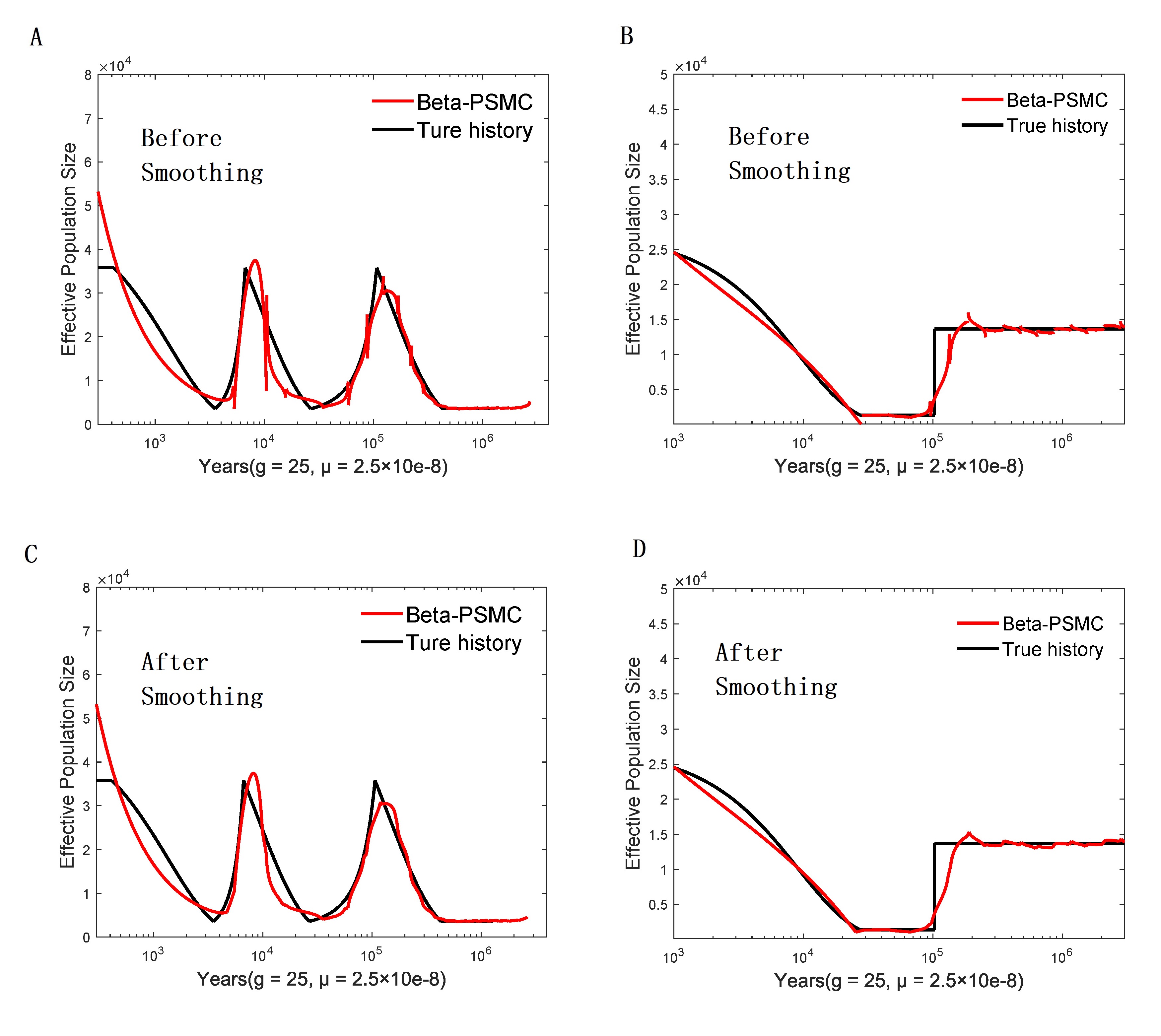


Fig. S5. Smoothing the connections between adjacent time intervals to improve the inference of demographic history. g, generation time; μ, mutation rate. The number of subintervals for each time interval is 3.

**References**

Li, C. et al. (2014) Two Antarctic penguin genomes reveal insights into their evolutionary history and molecular changes related to the Antarctic environment. GigaScience, 3.
